# Thyroid Swelling and Thyroiditis in the Setting of Recent hCG Injections and Fine Needle Aspiration

**DOI:** 10.1155/2016/2915816

**Published:** 2016-01-28

**Authors:** Elizabeth M. Lamos, Kashif M. Munir

**Affiliations:** Division of Endocrinology, Diabetes and Nutrition, University of Maryland School of Medicine, 827 Linden Avenue, 2nd Floor, Baltimore, MD 21201, USA

## Abstract

A 60-year-old woman presented with a neck mass and underwent fine needle aspiration of a left thyroid nodule. During this time, she had been injected with hCG for weight loss. Soon after, she developed rapid diffuse thyroid growth with pain. She was ultimately diagnosed with thyrotoxicosis due to postaspiration subacute thyroiditis and subsequently became hypothyroid. This condition is rare in the nonpregnant state in noncystic nodules with a smaller needle gauge approach. The incidence of thyroid nodule discovery and evaluation is increasing. As more procedures are undertaken, understanding of potential complications is important. This case highlights potential complications of thyroid fine needle aspiration including diffuse thyroid swelling and thyroiditis. The role of hCG injections is speculated to have potentially stimulated thyroid follicular epithelium via cross-reactivity with the TSH receptor and contributed to the acute inflammatory response after fine needle aspiration.

## 1. Introduction

The incidence of thyroid nodule discovery and evaluation is increasing. Fine needle aspiration biopsy (FNA) is the preferred procedure to evaluate for malignant potential of a thyroid nodule. There are multiple sonographic criteria which indicate whether thyroid nodules should be evaluated by FNA. These include size, growth, echogenicity, border, vascularity, and calcifications. As more procedures are undertaken, understanding of potential complications is important. Typically, the recommendation is to hold anticoagulant medications, such as aspirin, warfarin, nonsteroidal anti-inflammatory medications, prior to FNA if possible to reduce the risk of bleeding or bruising. However, there are no medications that are specifically contraindicated while performing FNA.

Thyroiditis and thyroid swelling are uncommon adverse effects of FNA. The potential release of thyroglobulin, thyroid hormone stores, and cytokines can trigger a series of symptoms that can be associated with pain, tenderness, and symptoms of thyrotoxicosis. Nodule characteristics and needle bore size may be risk factors for developing thyroiditis after FNA. It is unknown whether pregnancy or elevated levels of hCG increase the risk of post-FNA thyroiditis. FNA is generally a safe procedure; however, serious complications can occur. There is likely an underestimation of complications for such a common procedure. This case will highlight post-FNA thyroiditis and thyroid swelling potentially related to concurrent use of hCG injections.

## 2. Case Presentation

A 60-year-old woman presented to her primary care doctor with a newly discovered neck mass. A neck ultrasound demonstrated an enlarged thyroid with multiple bilateral nodules. Her TSH was 1.63 *μ*UI/mL and she was diagnosed with nontoxic multinodular goiter. Four weeks prior to noticing her neck enlargement, she had started taking daily subcutaneous urinary hCG injections (hCG) for weight loss. These injections were continued for 2 more weeks, for a total of 6 weeks ranging in dose from 175 to 200 IU daily.

**Figure 1:**
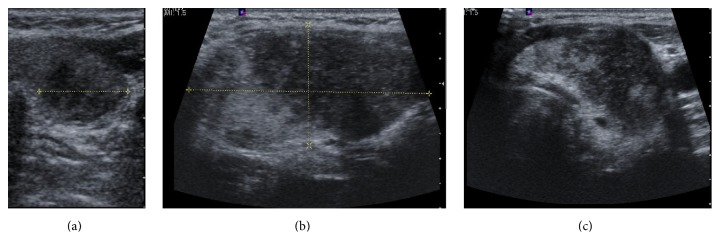
Thyroid ultrasound: (a) left midlobe dominant nodule in transverse view 1.4 × 1.3 × 1.5 cm (total lobe volume 8 mL) before FNA; (b) left lobe nodule in sagittal view 3.9 × 2.3 × 2.2 cm (total lobe volume 15.5 mL) after FNA; (c) left thyroid lobe 1 month after second FNA (total lobe volume 6.5 mL).

**Figure 2:**
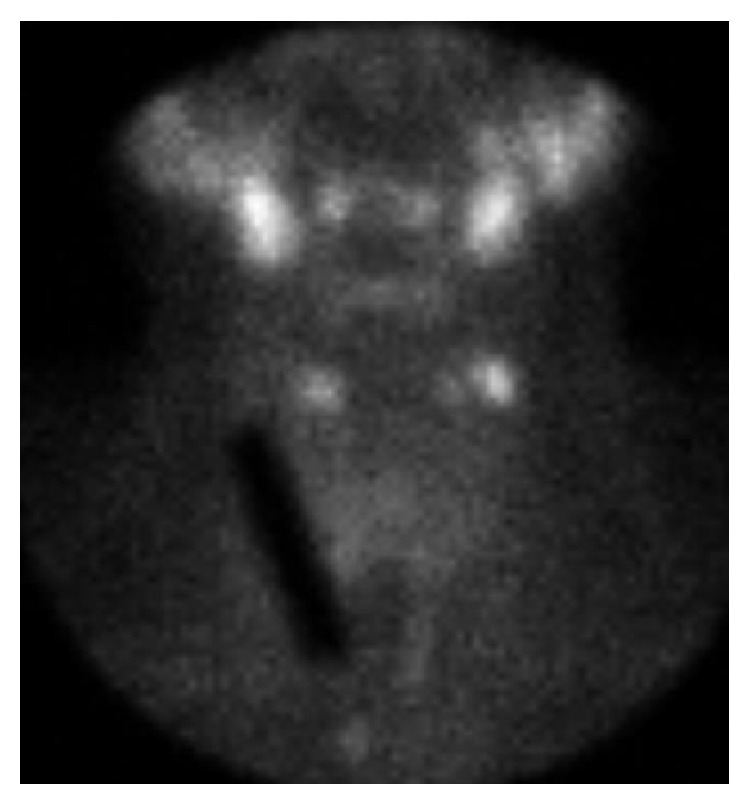
I-131 thyroid scan at 24 hours. Markedly diminished radiotracer accumulation is seen throughout the gland. The photopenic parallelogram over the right neck area is due to a 1 cm × 5 cm lead marker placed over the skin for size estimation purposes.

About one month after her initial ultrasound, she underwent FNA of the dominant left solid/complex lower pole nodule measuring 1.4 × 1.3 × 1.5 cm with 4 passes using 25 gauge needles (Figure 1(a)). The initial biopsy procedure was performed without complication. The biopsy was consistent with an adenomatous nodule consisting of benign follicular epithelial cells, few Hurthle cells, and scant colloid. Within one month of the unilateral aspiration, she reported neck enlargement, pain, and dysphagia. An esophagogram was normal. A repeat thyroid ultrasound demonstrated a >100% increase in overall goiter volume. Two new large nodules were identified, a right 5.1 × 1.9 × 2.7 cm hypoechoic, vascular lesion essentially replacing the right lobe and a left midlobe 3.9 × 2.3 × 2.2 cm heterogeneous, irregular, hypervascular lesion (Figure 1(b)). Concern for a rapidly growing neck mass obligated repeat FNA. Bilateral FNA demonstrated normal follicular cells in a background of inflammatory cells consistent with benign nodules in the setting of lymphocytic thyroiditis. TSH was suppressed at 0.03 *μ*IU/mL (0.34–5.6 *μ*IU/mL), free T4 was elevated at 2.07 ng/dL (0.58–1.64 ng/dL), and thyroid peroxidase antibody was elevated at 148 IU/mL (nl < 34 IU/mL). An I-131 thyroid uptake resulted in values as low as 1.8% and 2.3% at 2 and 24 hours, respectively, and the thyroid scan obtained at 24 hours showed significantly diminished tracer accumulation throughout the entire gland (Figure 2). Both the uptake values and the scintigraphic appearance were consistent with thyroiditis. During the period of acute inflammation, the patient experienced palpitations, tachycardia, and severe anterior neck pain. Metoprolol XL 25 mg daily and ibuprofen three times daily provided symptomatic relief for 2 weeks and then were discontinued. The neck swelling and pain continued to improve over the next month with conservative measures.

Within the following month, a repeat thyroid ultrasound demonstrated a 40% volume reduction in the overall size of the right lobe and a 60% volume reduction of the left lobe (Figure 1(c)). The entire gland echotexture and vasculature were heterogeneous and no discrete nodules were visible. The serum TSH remained suppressed at 0.03 *μ*IU/mL, but the free T4 was normal at 0.82 ng/dL and the TT3 was normal at 1.0 ng/mL (0.87–1.78). Subsequently TSH rose to 10.3 *μ*IU/mL after 6 weeks, and she was initiated on thyroid hormone replacement therapy.

## 3. Discussion

FNA of thyroid nodules is a common in-office or outpatient procedure to evaluate for malignancy. It is generally well-tolerated and safe. The most common adverse events include pain and local bleeding or hematoma and rarely result in infection [1]. Case reports in the literature describe acute or delayed (24 hour) swelling of the thyroid gland during or after fine needle aspiration [2]. The cases describe unilateral FNA but subsequent subacute (~4 weeks after procedure) diffuse gland swelling. In all cases but one, a 22-gauge needle or larger was used for FNA. This is in contrast to our case in which a smaller diameter 25 gauge needle was used. All cases were quick in onset and transient and in only one case were corticosteroids administered. Post-FNA thyroiditis is rare. One study demonstrated an incidence of 1% of the patients who underwent FNA [3]. These individuals had a higher cystic component to the nodule that was aspirated. The mechanism for the thyroiditis is suspected to be leakage of cystic material triggering an inflammatory response and release of preformed hormone resulting in a thyroiditis. In our case, the patient underwent unilateral biopsy of a nodule that was complex but not primarily cystic. She developed bilateral swelling subacutely which was self-limited. In addition, both biochemically and radiologically, she had coexisting thyroiditis. As discussed above, both thyroid swelling and subacute thyroiditis are rare after FNA and occurrence simultaneously within the same nonpregnant patient has not been previously described in the literature to our knowledge.

The role of hCG injections in provoking thyroiditis is unclear. hCG is a hormone produced during pregnancy. Its use in weight loss was popularized in the 1950s in conjunction with a very low calorie diet. Multiple studies have debunked the efficacy of hCG injections in weight loss as well as elucidated potential adverse effects like arrhythmias, gallstones, and harmful changes in sex hormones [4]. Subsequently, the FDA and the Endocrine Society recommend against this unapproved weight loss method. The dose of daily subcutaneous injections that is typically described in hCG diets does not appear to meet the physiologic serum concentration that would be seen, for example, in pregnancy or trophoblastic tumors. However, pharmacokinetic parameters of multiple daily injections for up to 6 weeks (as with this patient) in the dosing described above have never been evaluated [5]. As the alpha subunit of hCG is homologous to the TSH alpha moiety, it is reasonable to consider that a low level of chronic stimulation to thyroid follicular cells could be occurring. This is seen in pregnancy whereby the elevated hCG results in benign hyperplasia and diffuse enlargement of the thyroid gland [6, 7]. This tonic stimulation could have primed the thyroid tissue in this patient and have been the underlying mechanism by which an inflammatory response occurred. Cytology alone may not be adequate to demonstrate follicular hyperplasia. However, a causal relationship between the development of thyroid swelling and subacute thyroiditis to either the hCG injections or the FNA cannot be proven.

## 4. Conclusion

As thyroid nodules are more commonly diagnosed and patients are undergoing routine FNA evaluation for malignancy, practitioners should be aware of the acute but rare complications of thyroid swelling and thyroiditis. Avoiding larger needle gauge and consideration of the appropriateness of aspiration of essentially cystic nodules has been described before. However, use of smaller gauge needles and nodules with more complex characteristics should neither comfort the individual attempting biopsy nor delay the diagnosis or treatment of such a complication. Additionally, a rapidly growing neck mass can be concerning for lymphoma or aggressive thyroid malignancy. Although self-limiting, thyroid swelling and thyroiditis can result in a rapidly expanding neck mass which can be stressful to both the patient and practioner. The mechanism behind this inflammatory response is unknown but this case suggests that hCG administration could be one mechanism by which the thyroid parenchyma could be primed for an inflammatory response.
